# Influence of Anodization Temperature on Geometrical and Optical Properties of Porous Anodic Alumina(PAA)-Based Photonic Structures

**DOI:** 10.3390/ma13143185

**Published:** 2020-07-16

**Authors:** Ewelina Białek, Maksymilian Włodarski, Małgorzata Norek

**Affiliations:** 1 Institute of Materials Science and Engineering, Faculty of Advanced Technologies and Chemistry, Military University of Technology, Str. gen Sylwestra Kaliskiego 2, 00-908 Warsaw, Poland; ewelina.bialek2@gmail.com; 2 Institute of Optoelectronics, Military University of Technology, Str. gen. Sylwestra Kaliskiego 2, 00-908 Warsaw, Poland; maksymilian.wlodarski@wat.edu.pl

**Keywords:** porous anodic alumina (PAA), pulse anodization, distributed Bragg reflector (DBR), PAA-based DBR, transmission spectra, photonic stop band (PSB), temperature

## Abstract

In this work, the influence of a wide range anodizing temperature (5–30 °C) on the growth and optical properties of PAA-based distributed Bragg reflector (DBR) was studied. It was demonstrated that above 10 °C both structural and photonic properties of the DBRs strongly deteriorates: the photonic stop bands (PSBs) decay, broaden, and split, which is accompanied by the red shift of the PSBs. However, at 30 °C, new bands in transmission spectra appear including one strong and symmetric peak in the mid-infrared (MIR) spectral region. The PSB in the MIR region is further improved by a small modification of the pulse sequence which smoothen and sharpen the interfaces between consecutive low and high refractive index layers. This is a first report on PAA-based DBR with a good quality PSB in MIR. Moreover, it was shown that in designing good quality DBRs a steady current recovery after subsequent application of high potential (U_H_) pulses is more important than large contrast between low and high potential pulses (U_H_-U_L_ contrast). Smaller U_H_-U_L_ contrast helps to better control the current evolution during pulse anodization. Furthermore, the lower PSB intensity owing to the smaller U_H_-U_L_ contrast can be partially compensated by the higher anodizing temperature.

## 1. Introduction

Porous anodic alumina (PAA) is a multifunctional porous ceramic coating prepared by anodization of aluminum. Its geometrical parameters, such as interpore distance (*D_c_*) and pore diameter (*D_p_*), can be controlled by electrochemical conditions including type and concentration of electrolyte, temperature, applied voltage, and anodization time [1,2]. PAA with long-range hexagonally ordered and parallel pores is usually formed under self-ordering regimes, which are defined by narrow process windows characteristic for a given electrolyte [3,4]. Out of these regimes, the pore arrangement strongly deteriorates. The best hexagonal pore ordering is, however, obtained upon anodization conducted close to the so-called critical voltage, where high current/high electric field strength conditions are operative [5,6]. The larger the dissociation constant for a given electrolyte, the lower is the critical voltage [7]. Stability of the anodization is regulated also by temperature: the higher the temperature, the lower the critical voltage. The applied voltage determines mostly the *D_c_* [8,9], while temperature—both *D_c_* and *D_p_* [10,11]. Porosity of PAA is related to both interpore distance and pore diameter via the equation: $P=\frac{\pi }{2\sqrt{3}}{\left(\frac{D_{p}}{D_{c}}\right)}^{2}$ [12]. PAA can be used as a template to fabricate various functional nanostructures [13,14,15] or itself is a functional material used in gas separation [16], medicine (tissue engineering) [17], or electronics (supercapacitors) [18]. One of the recent applications of PAA is related with a possibility to engineer PAA-based photonic structures.

The photonic structures based on porous materials are usually one-dimensional (1D) photonic crystals (PCs) built of many alternating low and high refractive index (RI) layers and the thickness adequate to obtain the enhancement of a selected wavelength (*λ*), as a result of constructive interference of waves reflected from the interface between the neighboring layers (the photonic stopband, PSB) [19,20]. The PSB is thus related to the wavelength ranges where the material demonstrates high reflectivity and low transmittance of light. The refractive index of porous layer is strictly related with its porosity (*P*): RI decreases as *P* increases [21]. Therefore, one of the important issue in designing this type of photonic crystals is a precise control over the porosity of the alternating and subsequent layers. The larger the refractive index contrast (the difference between the low and high RI of alternating layers), the more light is reflected from the layer boundary, and the more intensive PSB at a given λ can be obtained. Spectral position of PSB is very sensitive to a slight change of refractive index of a medium, and this property is a base for engineering a broad range of optical sensors [22,23,24,25].

To obtain PAA with periodically variable porosity of alternating layers, pulse anodization is applied which relies on periodic change of anodization parameters, such as voltage or current density [26,27]. The pulse sequence can be modified accordingly (different anodization modes, different current/voltage values of the generated cycles, various shape of the pulses including saw-like or pseudo-stepwise anodization waves, various ramp rate between high (U_H_) and low potential (U_L_), different duration of the pulses, variable number of pulses, etc.) to design a desired pore architecture and to mold strong and narrow resonances at a given spectral range. During pulse anodization other factors—such as electrolyte composition, concentration, and temperature—need to be carefully selected in order to maintain the self-ordering conditions without uncontrolled pore branching, or burning effects. PAA-based PCs developed so far were synthesized prevalently in sulfuric [28,29,30,31,32] and oxalic [33,34,35,36] electrolytes, using galvanostatic (current density control) or potentiostatic (voltage control) mode. First pulse anodization experiments in oxalic acid appeared to be quite challenging as compared to those in sulfuric acid owing to a relatively dense and compact barrier layer formed under high potential pulses (U_H_ = 55 V) [37]. The dense barrier layer prevented passage of ions under application of subsequent low potential pulses (U_L_ = 40 V) [37]. However, it was later shown that a uniform growth of alternating low and high RI layers is also possible upon application of a stepwise decrease of U_H_ to U_L_, which allowed for a continuous barrier layer thinning [34]. Moreover, thanks to the application of a suitable voltage ramp rate, it was possible to increase the contrast between the U_H_ (50 V) and U_L_ (20 V) potentials, which translates into the larger refractive index contrast. Since then the influence of various pulse profiles [38,39], durations and amplitudes [40] on PAA-based PCs architecture and optical properties was studied. The optical properties of PAA-based photonic crystals were also tuned via application of mixed oxalic and phosphoric acid concentrations [41]. Mixing different concentration of phosphoric acid (0.05, 0.1, 0.2, and 0.3 M) with 0.3 M oxalic acid solution has allowed to expand the amplitudes of the applied voltage pulses (up to U_HA_ = 100 V) and to improve the PSB signal quality (smaller full width at half maximum, FWHM, and greater intensity). Despite quite extensive research on the impact of various parameters on optical properties of PAA-based PCs, little attention was paid so far to anodizing temperature, although it is a very important factor governing the growth rate of PAA layers. Furthermore, it is expected that apart from increasing the reaction speed other parameters, such as pore regularity and circularity, will be improved at higher temperatures [10]. This, in turn, should have a beneficial effect on the quality of PSBs. The photonic properties of PAA-based photonic structures were previously studied in a narrow temperature range (6–18 °C in [42] and 8–11 °C in [43]). However, it is of great interest to systematically study the impact of temperature on the properties of PAA-based PCs in a much broader range.

In this work, we analyze the impact of temperature on the quality of the PAA-based distributed Bragg reflector (DBR) structure fabricated in oxalic electrolyte in the temperature range 5–30 °C. The impact of temperature on spectral position and intensity of PSB is systematically analyzed. It is shown that in this temperature range the photonic properties of PAA-based DBR change immensely. The PSB resonances worsen at the temperature >10 °C: the resonance peaks in transmission spectra fade away, broaden, and split, which is accompanied by the red shift of PSBs. However, at 30 °C, the peaks appear again with a strong and symmetric PSB in the mid-infrared (MIR) spectral region. Moreover, the photonic properties in the MIR are improved by a small modification of pulse sequence which sharpens the boundaries between low and high RI segments in PAA. This is a first report on production of PAA-based DBR with a good quality PSB in MIR. The results obtained in this work can extend the application of the PAA-based photonic structures up to the MIR spectral range.

## 2. Materials and Methods

The PAA-based distributed Bragg reflector (DBR) structures were synthesized by a pulse anodization of aluminum. High-purity aluminum foil (99.9995% Al, Puratronic, Alfa-Aesar, Haverhill, MA, USA) with a thickness of about 0.25 mm was cut into specimens (2 cm × 1 cm). Before the anodization process the Al foils were annealed under argon atmosphere at 400 °C for 2 h. Then the samples were degreased in acetone and ethanol and subsequently electropolished in a 1:4 mixture of 60% HClO_4_ and ethanol at 0 °C, under constant voltage of 25 V, at 1 °C, and for 2.5 min. Next, the samples were rinsed with a distilled water, then ethanol, and dried. As prepared Al specimens were insulated at the back and the edges with acid resistant tape, and serve as the anode. A Pt grid was used as a cathode and the distance between both electrodes was kept constant (ca. 5 cm). A large, 1 L electrochemical cell, a powerful low-constant-temperature bath, and vigorous stirring (500 rpm) were employed in the anodizing process. Programmable DC power supply, model 62012P-600-8 Chroma, was used to control the applied voltage and the pulse parameters. The first anodization was carried out at 5 °C in 0.3 M C_2_H_2_O_4_ water-based solution, at 40 V, for 20 h. As obtained alumina was chemically removed in a mixture of 6 wt % phosphoric acid and 1.8 wt % chromic acid at 60 °C for 3 h. Subsequently, pulse anodization with 20 cycles was conducted at the temperature range 5–30 °C. In general, a pulse cycle consisted of three steps: (1) a constant high voltage step (U_H_ = 50, 45, 40 V) applied for 360 s; (2) a gradual reduction of the voltage to 20 V (and to 30 V) at rates of 0.312, 0.234, 0.156, and 0.078 V/s (and at 0.07 V/s, respectively); (3) the anodization at a constant low voltage (U_L_ = 20 V) for 480 s (one sample was anodized under U_L_ = 30 V and the pulse duration of 3600 s). After the pulse anodization was completed, the remaining aluminum substrate was selectively removed in a saturated solution of HCl/CuCl_2_.

Structural characterization of the PAA-based photonic structures was made using a field-emission scanning electron microscope FE-SEM (AMETEK, Inc., Mahwah, NJ, USA) equipped with energy dispersive X-ray spectrometer (EDS). The measurement of layer thickness was repeated three times at different points in the image of a given PAA sample and an average of the three measurements was taken to determine the initial and final d_H_ and d_L_ thickness and the total thickness of the PAA membrane (d_tot_).

The transmission spectra were measured with two instruments. Shortwave end of the spectrum (250–2500 nm) was measured by Cary 5000 spectrometer with DRA-2500 integrating sphere from Agilent Technologies Inc., Santa Clara, CA, USA. Longwave end of the spectrum (2500–25,000 nm) was measured by Fourier-transform infrared (FTIR) spectrometer Alpha II from Bruker Corp., Billerica, MA, USA. The resolution of spectra was set to 1 nm in shortwave range and 2 cm^−1^ in longwave range.

## 3. Results and Discussion

**Figure 1:**
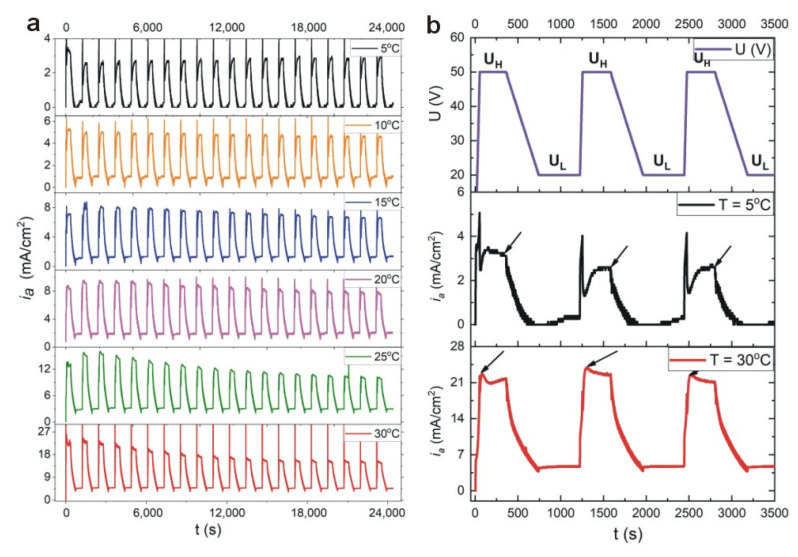
Current density (*i_a_*)—time (*t*) transients during pulse anodization (20 cycles) of aluminum at temperatures 5–30 °C (**a**); first three U_H_-U_L_ pulses along with the *i_a_*(*t*) curves for anodization at 5 °C and 30 °C (**b**). The black arrows in Figure 1b signify the *i_a_^max^*: depending on the type of anodization process (MA or HA) the *i_a_^max^* value was determined at the end or the beginning of the U_H_ pulse.

Current density (*i_a_*)—time (*t*) transients during pulse anodization (20 cycles) of aluminum at temperature (T) between 5–30 °C are shown in Figure 1a. The PAA-based DBR is formed by applying a series of potential pulses, comprising high potential pulse (U_H_ = 50 V and t_H_ = 360 s) followed by a low potential pulse (U_L_ = 20 V and t_L_ = 480 s). First three pulse profiles (U(V)) together with *i_a_*(*t*) curves recorded at 5 °C and 30 °C are demonstrated in Figure 1b. It can be seen that the current characteristics changes with temperature. At relatively low temperatures (5–15 °C), after application of U_H_ pulse, the current recovery effect is typical for conventional mild anodization (MA) processes, where current is large at the initial stage, goes to a minimum value passing through a current pike, and then increases gradually to reach a steady value. However, at higher temperature (20–30 °C) upon applying the U_H_ pulse, the current increases steeply for a short period of time and then decreases exponentially. The latter behavior is typical for hard anodization (HA) processes. The current recovery peak (*i_a_^max^*) after application of the successive U_H_ pulses starts to decay visibly for the samples anodized at T >10 °C. At 30 °C, the last current recovery peak is about 30% less intensive than the first one.

**Figure 2:**
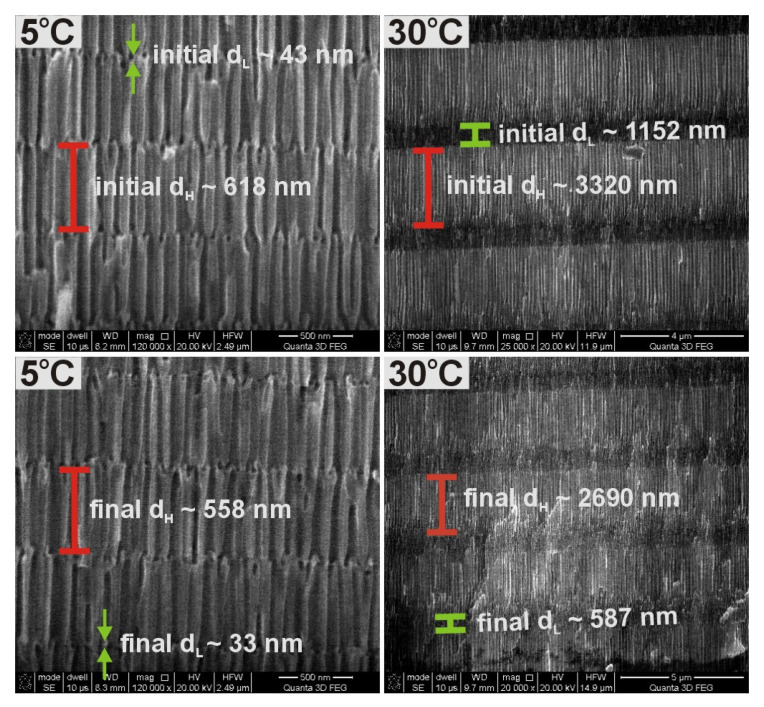
SEM images of a cross sectional view of the initial and final PAA segments obtained during the pulse anodization at 5 °C (the sample PAA—5 °C) and 30 °C (the sample PAA—30 °C).

The unequal current recovery indicates that the total amount of charge in each U_H_ pulse decreases as the number of pulses increases. Since the thickness of anodic alumina is directly proportional to the net amount of charge involved in anodization reaction, it is thus expected that anodic alumina formed under the present conditions will contain segments with non-uniform thickness. Thickness of three initial and final segments (corresponding with the first and last potential pulses in the 20-cycle anodization process) formed under U_H_ and U_L_ potentials (d_H_ and d_L_, respectively) for PAA produced at the two extreme temperatures is presented in Figure 2. As can be seen, the difference between initial and final d_H_ and d_L_ segments is much larger in the PAA-based DBR formed at 30 °C as compared to that formed at 5 °C.

**Figure 3:**
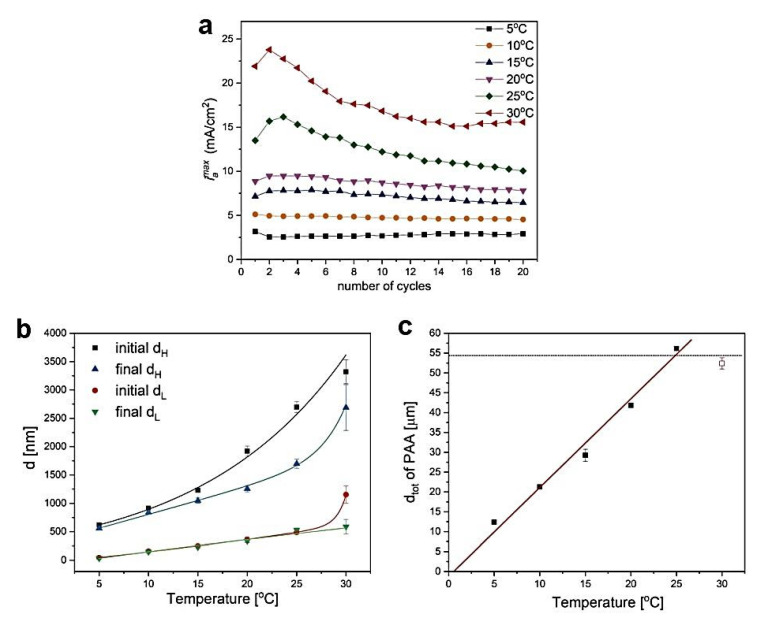
*i_a_^max^* as a function of the number of cycles (**a**) thickness of initial and final d_H_ and d_L_ segments as a function of anodizing temperature (**b**) total thickness (d_tot_) of PAA membranes as a function of anodization temperature (**c**).

The evolution of *i_a_^max^* (indicated by black arrows in Figure 1b) after application of the following 20 U_H_ pulses for all samples anodized at temperatures 5–30 °C is well visible in Figure 3a. At 5 °C and 10 °C, the *i_a_^max^* remains more or less at stable values during each cycle of anodizing, however, starting already from 15 °C the *i_a_^max^* gets weakened noticeably as the number of U_H_ pulses increase. At 25 °C and 30 °C, the *i_a_^max^* intensity drop is very significant. The behavior is followed by the increasing difference between initial and final d_H_ and d_L_ layers (Figure 3b). First of all, an increase of temperature results in thicker d_H_ and d_L_ slabs owing to the enhanced electrochemical reaction rate. The d_L_ difference is rather negligible in the temperature range 5–25 °C but starts to be pronounced at 30 °C. However, the difference between first and last d_H_ segments begins to grow substantially already at T >10 °C. The effect indicates the appearance of diffusional problems at the higher temperatures related with the extended diffusion path along the nanopores and consequently slower mass transport (the oxygen-containing anionic species such as O^2−^, OH^−^) from the electrolyte to the pore bottom [44,45,46]. Nevertheless, the total thickness (d_tot_) of PAA dependence on temperature (in the 5–25 °C range) is almost linear (Figure 3c) what suggests that other, rate-limiting processes have to be also accounted for this behavior. The d_tot_ of PAA prepared at 30 °C is an exception here. The sudden collapse of the linear relationship for this sample could indicate the existence of a boundary thickness (around 54 μm), above which Al_2_O_3_ stops to grow.

**Figure 4:**
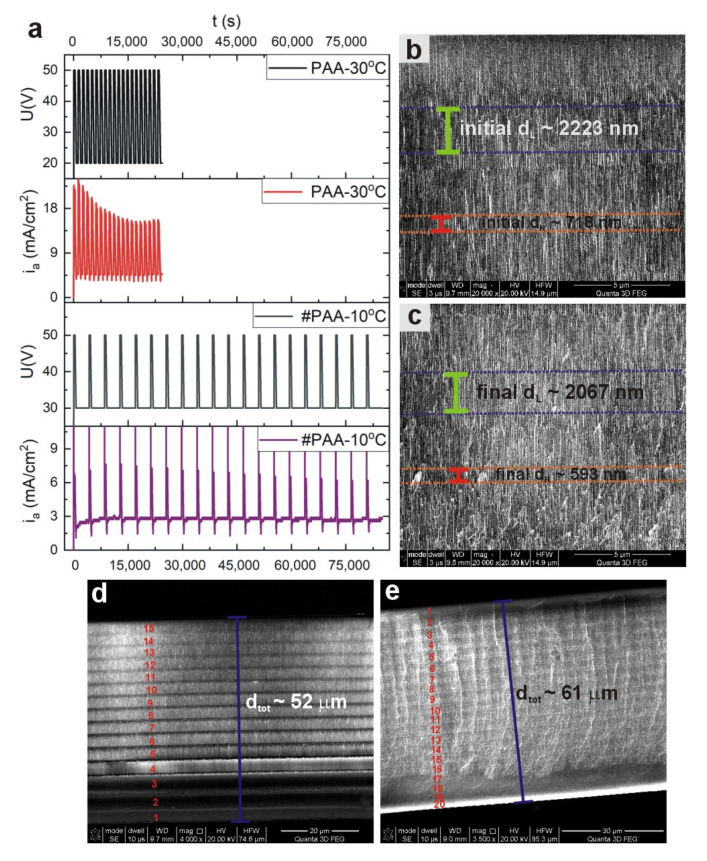
*i_a_*(*t*) curves recorded during pulse anodization (20 cycles) of the sample PAA—30 °C (U_H_ = 50 V with t_H_ = 360 s, U_L_ = 20 V with t_L_ = 480 s. at 30 °C) and #PAA—10 °C (U_H_ = 50 V with t_H_ = 360 s, U_L_ = 30 V with t_L_ = 3600 s. at 10 °C) (**a**); SEM images of initial (**b**) and final (**c**) d_H_ and d_L_ segments in the sample #PAA—10 °C; SEM image of a cross-sectional view of the whole PAA—30 °C (**d**) and #PAA—10 °C (**e**) membranes.

To get deeper insight into this issue, additional PAA-based DBR structure under the following anodizing conditions was prepared: U_H_ = 50 V with t_H_ = 360 s, and U_L_ = 30 V with t_L_ = 3600 s. In order to avoid too extensive PAA growth, the process was conducted at 10 °C (therefore the sample will be further named as #PAA—10 °C). The *i_a_*(*t*) curves recorded during anodization of #PAA—10 °C and PAA—30 °C samples are compared in Figure 4a. Despite prolonged anodization time of the #PAA—10 °C sample (the whole process lasted ca. 24 h), the *i_a_^max^* remains at quite stable values after application of subsequent U_H_ pulses (the recovery of current after application of the U_L_ pulses is very regular), in contrast to the *i_a_^max^* recorded during pulse anodization of the sample PAA—30 °C. As an effect of the steady current evolution, the thickness of initial and final d_H_ and d_L_ segments in the sample #PAA—10 °C does not differ so much as in the sample PAA—30 °C (Figure 4b,c). Particularly, the thickness of initial d_L_ layers is larger only of about 7% from that of the final ones. Closer examination of the sample PAA—30 °C revealed that it is built only out of 15 d_L_ and d_H_ layers instead of 20, which explains its lower d_tot_ (~52 μm) than expected (Figure 4d). The resulted d_tot_ of the #PAA—10 °C is ca. 9 μm thicker (d_tot_~61 μm) than the PAA—30 °C (Figure 4e). Moreover, despite the larger thickness of the membrane, the #PAA—10 °C photonic crystal consists of full 20 d_L_ and d_H_ layers (Figure 4e). It is therefore evident that the growth of the remaining 5 d_H_ and d_L_ layers in the PAA—30 °C sample was not stopped solely by diffusional problems related with too thick membrane. Moreover, successful formation of the 15 subsequent t_H_ and t_L_ layers in PAA—30 °C DBR indicates that the mass transport (movement of ionic species, such as O^2−^, OH^−^, Al^3+^ through the barrier layer) was not hindered by a too thick barrier layer formed at the high temperature. On the contrary, the high temperature provided sufficient driving force to overcome this barrier. Most probably a high reaction rate, which requires a continuous and relatively fast delivery of the anionic species from the bulk reservoir to the pore basis (from the electrolyte to the oxide/metal interface), was mainly responsible for reaction termination during pulse anodization at 30 °C. The electrochemical formation of PAA is determined by both diffusion-controlled and rate-controlled processes. If the PAA thickness is increased too much, the delivery is substantially delayed, and the electrochemical reaction at 30 °C is terminated due to the high reaction speed. In the case of the sample #PAA—10 °C the reaction rate is slowed down so much that the larger distances that ions have to overcome along the increasing thickness of PAA does not constitute an obstacle in the oxide formation.

**Figure 5:**
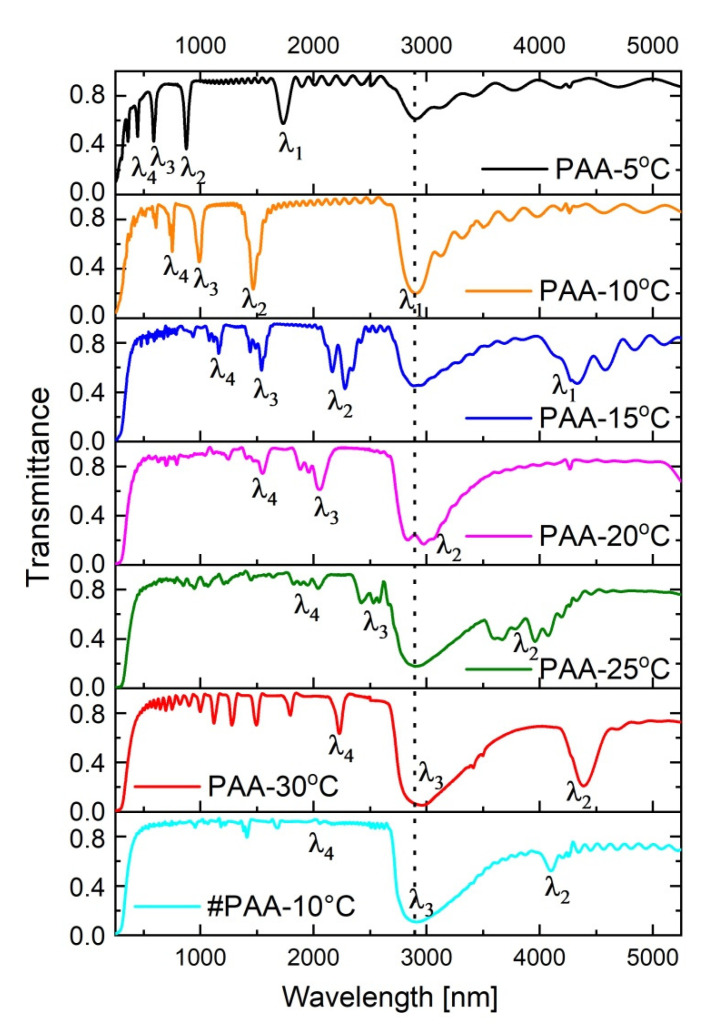
Transmittance spectra of PAA-based photonic structures anodized at temperature range 5–30 °C (the broad peak at around 3000 nm, present in all spectra and marked by vertical, black, dotted line, originate from OH group vibrations of adsorbed water [49]).

In Figure 5, transmission spectra of the PAA-based DBR structures fabricated at the temperature range 5–30 °C are presented. The position of photonic stop bands (PSBs) is usually determined based on Bragg–Snell law [47]

(1) $$m\lambda =2d\sqrt{n_{eff}^{2}-n_{air}^{2}sin^{2}\theta }$$

where *λ* is the wavelength of a stop band, *m* is the order of the PSB, *d* is the layer thickness (periodicity), *θ* is the angle of incidence, *n_eff_* is the effective refractive index, and *n_air_* is the refractive index of air.

In the transmission spectra (T(λ)) several resonance peaks are visible which can be assigned to different orders of a given stop band (λ_i_, i = 1–4, correspond to 1–4 orders of PSB; the bands were assigned to the λ_i_ based on the Bragg–Snell equation, assuming *θ*~0). At temperatures 5 °C and 10 °C, the peaks are distinct and narrow. As compared to the PSBs in the sample PAA—5 °C, the peaks in the spectrum of the sample PAA—10 °C are more intensive and red-shifted. Upon increasing temperature, the peaks shift further towards the red part of the spectrum, split, and become progressively broadened. In the spectrum of the PAA—25 °C they almost disappear. However, in the spectrum of the sample PAA—30 °C the peaks start to show up again: several low-order ones with lower intensity in the range 1000–2500 nm, and one located in the mid-infrared region, centered at ~4386 nm with T~0.20 (according to a commonly used subdivision scheme MIR region falls into 3–8 μm [48]). The stop band in the MIR region (assigned to λ_2_ of the PSB) is very symmetric, what usually indicates a good quality photonic crystal structure. Moreover, based on the Bragg–Snell equation (for *θ*~0) and taking into account the measured periodicity (*d* = d_H_ + d_L_) and λ_i_, the *n_eff_* of the studied PAA-based DBRs can be roughly estimated to be within the range of 1.13–1.57.

The spectrum of the # PAA—10 °C DBR is quite similar to the one of the sample PAA—30 °C in terms that it also shows several bands in the range 1000–2500 nm, and the one in the MIR region. The peaks are, however, much less intensive (the λ_2_ band is located at around 4100 nm with T~0.5).

The PAA—30 °C photonic crystal apparently has much better optical properties than the #PAA—10 °C crystal, despite its lower number of d_L_ and d_H_ layers and larger difference in the initial and final thickness of the d_L_ and d_H_ layers. This phenomena can be due, however, to a larger refractive index contrast (∆*n_eff_*) between the subsequent d_L_ and d_H_ segments. It was shown before that a stopband enlarges and sharpens when the ∆*n_eff_* increases [20]. The ∆*n_eff_* in PAA photonic material is directly related with porosity contrast (∆*P*) between the d_L_ and d_H_ layers [21], and the porosity, in turn, is determined by both anodizing voltage and temperature [10,11]: the larger the applied voltage and the higher the temperature the greater is the porosity. Therefore, the ∆*P* in the sample PAA—30 °C is tuned and increased by both larger U_H_-U_L_ contrast (30 V) and higher anodizing temperature (T = 30 °C) as compared to the lower U_H_-U_L_ contrast (20 V) and lower temperature (T = 10 °C) applied to fabricate the sample #PAA—10 °C.

**Figure 6:**
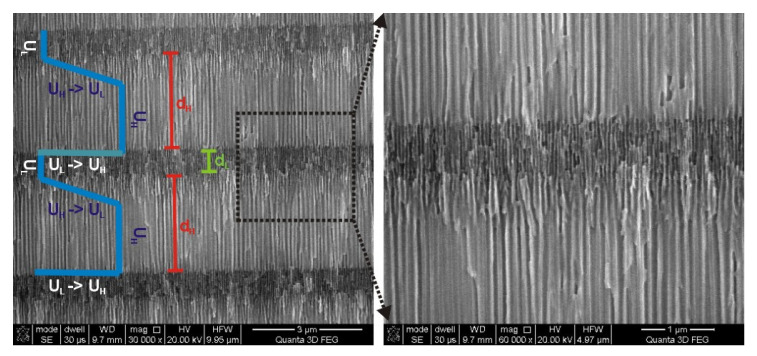
The U_H_ –˃ U_L_ and U_L_ –˃ U_H_ interfaces in the PAA—30 °C DBR.

**Figure 7:**
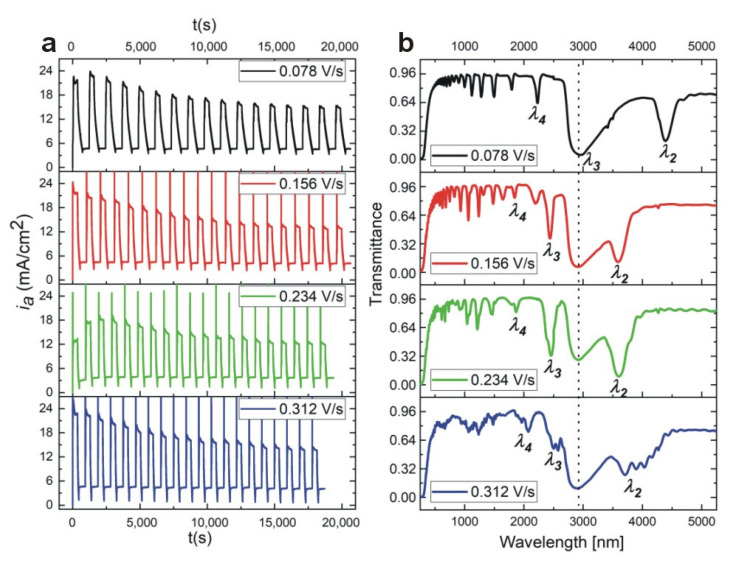
*i_a_*(*t*) curves recorded during pulse anodization (U_H_ = 50 V with t_H_ = 360 s, U_L_ = 20 V with t_L_ = 480 s, 20 cycles, at 30 °C) with decreasing U_H_ –˃ U_L_ drop rate form 0.078 V/s down to 0.312 V/s (**a**), the corresponding transmission spectra (the broad peak at around 3000 nm present in all spectra and marked by vertical, black, dotted line, originate from OH group vibrations of adsorbed water [49]) (**b**).

Morphology analysis of the PAA—30 °C DBR crystal suggests that its optical properties can be still improved by a better tailoring the d_H_ and d_L_ interfaces. In Figure 6, it can be noticed that the edge of the d_H_ segment that corresponds with the gradual decrease of voltage from U_H_ to U_L_ (U_H_ –˃ U_L_ edge) is very blurred as compared to the opposite edge that corresponds with a direct voltage change from U_L_ to U_H_ (U_L_ –˃ U_H_ edge). The clear and sharp interfaces between constitutive layers are known to be critical for highly reflective DBRs [50]. Therefore, the pulse sequence were modified accordingly by acceleration of the voltage drop rate in order to sharpen the U_H_ –˃ U_L_ edge. In Figure 7, *i_a_*(*t*) curves recorded during pulse anodization (U_H_ = 50 V with t_H_ = 360 s, U_L_ = 20 V with t_L_ = 480 s, 20 cycles, T = 30 °C) with increasing U_H_ –˃ U_L_ drop rate from 0.078 V/s up to 0.312 V/s are shown, along with the corresponding transmission spectra (the samples: PAA—30 °C_0.078, PAA—30 °C_0.156, PAA—30 °C_0.234, and PAA—30 °C_0.312, respectively).

It can be seen that upon decreasing the U_H_ –˃ U_L_ rate from 0.0718 V/s to 0.234 V/s the intensity of the corresponding transmission dips (λ_2_ and λ_3_) increases. The intensity of the *λ_2_* band in the PAA—30 °C_0.078 sample increases from 0.20 to 0.10 for the sample PAA—30 °C_0.156 and to 0.08 for the sample PAA—30 °C_0.234. At the same time, the *λ_2_* shifts from 4386 nm to 3587 nm and 3602 nm in the samples anodized with the lower U_H_ –˃ U_L_ rate (0.156 V/s and 0.234 V/s, respectively). Furthermore, the decrease of the U_H_ –˃ U_L_ rate to 0.312 V/s deteriorates the optical properties of the PAA—30 °C_0.312 DBR: the resonance peaks split and become hardly distinguishable.

**Figure 8:**
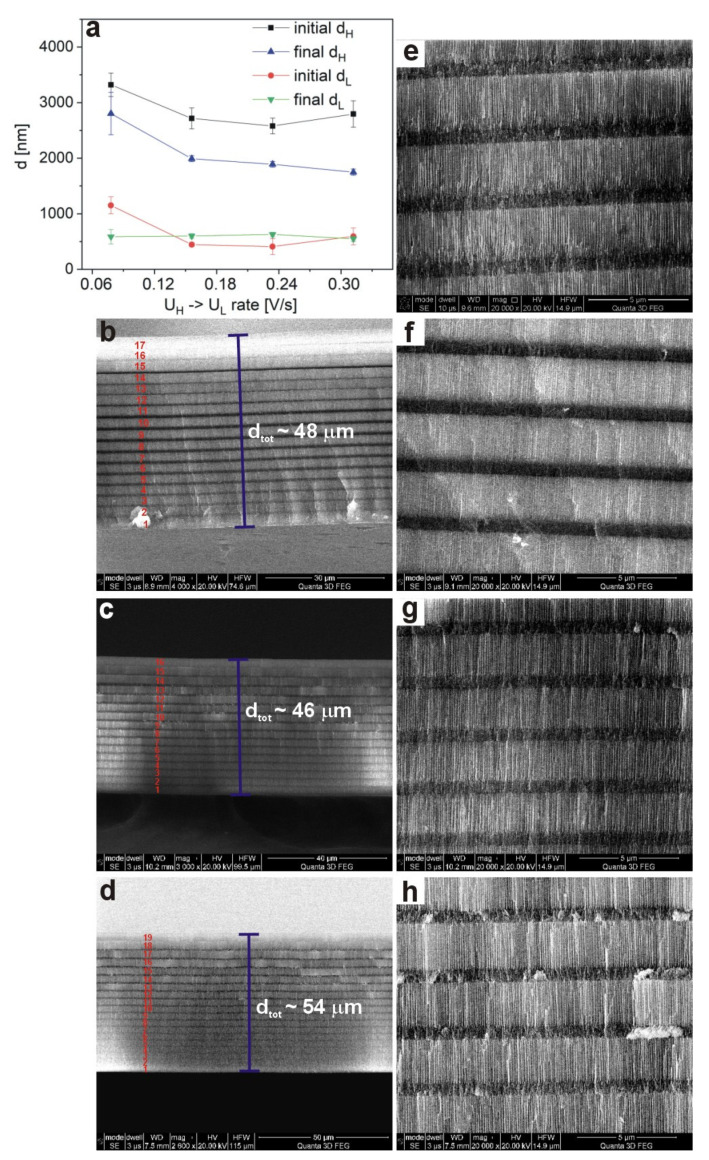
Thickness of d_H_ and d_L_ segments as a function of the U_H_ –˃ U_L_ drop rate (**a**); SEM images of a cross-sectional view of the whole PAA—30 °C_0.156 (**b**), PAA—30 °C_0.234 (**c**), and PAA—30 °C_0.312 (**d**) membrane; SEM images of middle d_H_ and d_L_ layers of the PAA—30 °C DBRs with U_H_ –˃ U_L_ rate of 0.078 V/s (**e**), 0.156 V/s (**f**), 0.234 V/s (**g**), and 0.312 V/s (**h**).

The shift of the *λ_i_* to shorter wavelength is presumably caused by the reduced thickness of both d_H_ and d_L_ layers formed in the PAA—30 °C DBR crystals prepared under the slower U_H_ –˃ U_L_ rates (Figure 8a). In Figure 8b–d, SEM images of cross-sectional views of the whole PAA membranes, prepared under various U_H_ –˃ U_L_ rates, are shown. It can be observed that the number of d_H_ and d_L_ segments increases as the U_H_ –˃ U_L_ rate decreases (Figure 8b–d). In the sample PAA—30 °C_0.312 almost all segments (19 out of 20) were formed. The d_tot_ varies also with the U_H_ –˃ U_L_ rate, but does not exceed 54 μm for the PAA—30 °C_0.312 sample. It seems thus that the 54 μm is indeed a limit thickness for PAA-based DBR grown at 30 °C. On the other hand, it can be expected that lowering T by few degrees (between 26 °C and 29 °C) will help to prepare DBR built out of full 20 d_H_ and d_L_ segments. This, in turn, will contribute to optimization of the photonic characteristics of PAA-based DBR with PSB resonances in MIR. Summarizing: the better optical quality of the samples PAA—30 °C_0.156 and PAA—30 °C_0.234 DBRs as compared to that of the PAA—30 °C_0.078 sample is caused by a larger number of constitutive d_H_ and d_L_ layers and by much sharper interfaces between d_H_ and d_L_ layers from the U_H_ –˃ U_L_ side (Figure 8e–h). Apparently, the transmission spectra of the PAA—30 °C_0.312 DBR deteriorates owing to the large difference between initial and final d_H_ thickness (Figure 8a), which eliminates potential benefits emerging from the largest number of subsequent d_H_ and d_L_ segments among all PAA-based DBRs fabricated at 30 °C.

**Figure 9:**
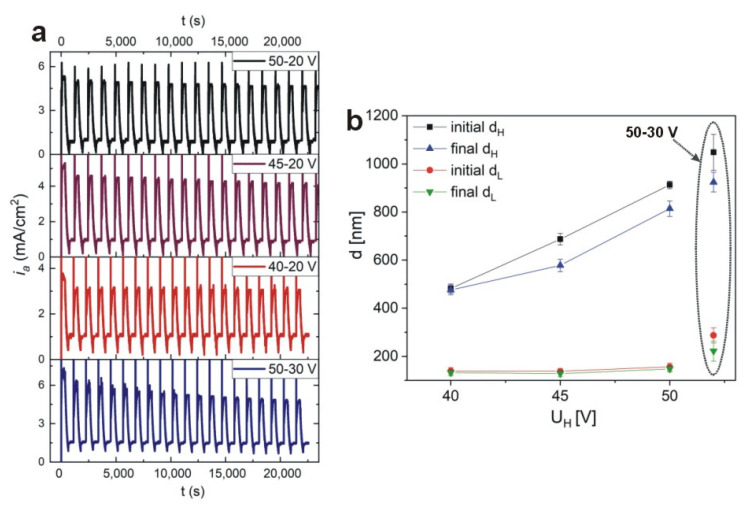
Current density *i_a_*(*t*) recorded during pulse anodization of aluminum under the following U_H_-U_L_ potentials: 50–20 V, 45–20 V, 40–20 V, and 50–30 V (t_H_ = 360 s, t_L_ = 480 s, 20 cycles, 10 °C) (**a**); initial and final d_H_ and d_L_ thickness as a function of U_H_ for the U_L_ = 20 V (the d_H_ and d_L_ thickness determined for the PAA—10 °C_50–30 sample is placed in the graph in a dotted ellipse) (**b**).

Based on transmission spectra in Figure 2, it can be stated that the strongest PSBs were generated in the PAA-based DBR fabricated at 10 °C. Therefore this temperature was selected to study the influence of U_H_ and U_L_ voltage on the optical properties of the PCs. In Figure 9a, the *i_a_*(*t*) curves for the DBRs synthesized under different values of U_H_ (50 V, 45 V, 40 V) and U_L_ (20 V, 30 V) are demonstrated, with other conditions kept as previously (the samples: PAA—10 °C_50–20, PAA—10 °C_45–20, PAA—10 °C_40–20, and PAA—10 °C_50–30, respectively). It can be observed that whereas the *i_a_^max^* decreases slowly with pulse cycles during anodization of the PAA—10 °C_50–20 and PAA—10 °C_45–20 DBRs, in the anodization of the PAA—10 °C_40–20 sample the *i_a_^max^* remains perfectly stable during all 20 cycles. The largest *i_a_^max^* drop is recorded for the PAA—10 °C_50–30 sample. The current evolution is reflected in the difference between initial and final thickness of d_H_ and d_L_ segments: for all PAA-based DBRs the difference is discernable, whereas in the DBR prepared under the 40–20 V all d_H_ and d_L_ layers are identical (Figure 9b). Particularly interesting is the comparison between the PAA—10 °C_40–20 and PAA—10 °C_50–30 samples. Despite the same U_H_-U_L_ contrast (20 V), the current behavior and the resulting DBR are quite different.

**Figure 10:**
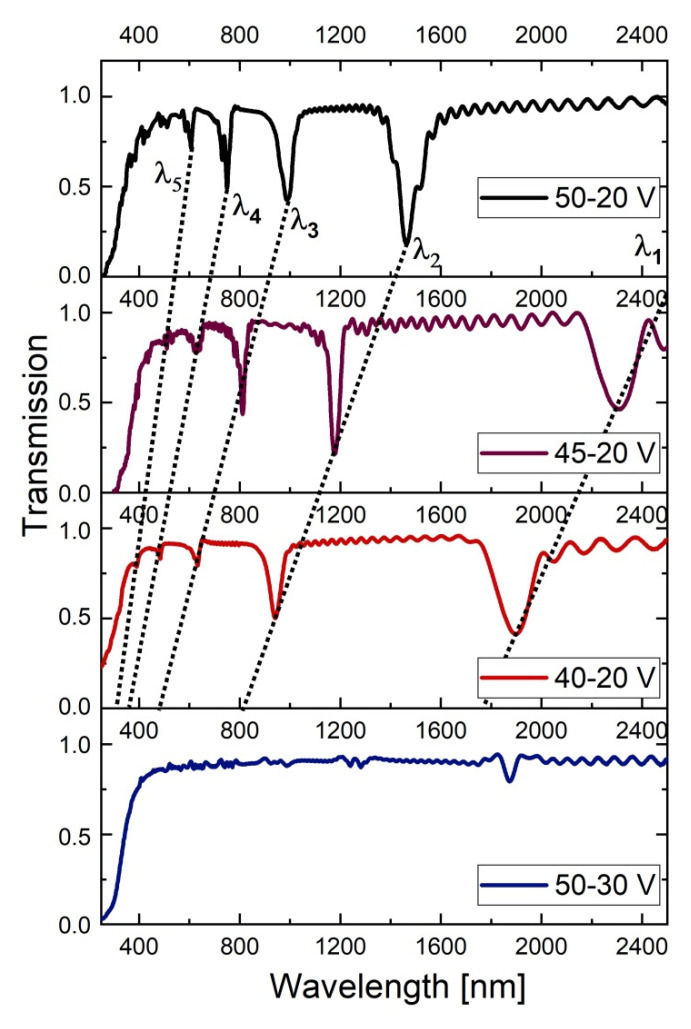
Transmittance spectra of PAA-based DBR fabricated at 10 °C under various U_H_ and U_L_ values.

The transmission spectrum of the PAA—10 °C sample is compared with T(λ) spectra of the other DBRs prepared at 10 °C in Figure 10, in the 250–2500 nm spectral range. First of all, upon decreasing U_H_ the PSBs are shifted towards blue part of the spectrum, mostly as an effect of decreasing the d_H_ layer thickness (Figure 9b). The intensity of the resonance peaks decreases progressively, however, the PSBs in the samples PAA—10 °C_45–20 and PAA—10 °C_40–20 become more symmetric and narrower as compared to the PSB in the sample PAA—10 °C_50–20. The narrower and more symmetric peaks, in turn, indicate better quality of the DBR crystals. In the T(λ) spectra of the PAA—10 °C_50–30 sample the PSBs have gone, meaning that basically no DBR structure was formed under this condition. Larger intensity of the resonance peaks in the T(λ) spectrum of the PAA—10 °C_50–20 DBR can be associated with a larger U_H_-U_L_ contrast (30 V) and consequently larger ∆*P*. However, owing to the larger U_H_-U_L_ contrast, the current recovery (*i_a_^max^*) after application of the following U_H_ pulses gets gradually weakened, and consequently, the formed d_H_ and d_L_ segments are not perfectly uniform throughout the whole PAA membrane. This, in turn, broadens the transmission peaks. As the U_H_-U_L_ contrast decreases (due to U_H_ decrease), the *i_a_^max^* becomes equalized (in the sample PAA—10 °C_40–20 the *i_a_^max^* is perfectly even) and the structural and optical properties of DBR are improved: the transmission peaks become narrower and more symmetric. However, the lower U_H_-U_L_ contrast makes the PSB peaks less intensive owing to the smaller ∆*P*.

Summarizing, when the U_H_ is lowered from 50 V to 40 V (while keeping the U_L_ constant) the quality of the crystals remains quite stable (or even improves). However, when the U_L_ values is increased from 20 V to 30 V (while keeping the U_H_ constant) the crystal quality is lost. The latter is caused by both small ∆*P* and the large *i_a_^max^* drop upon subsequent application of the U_H_ pulses. Based on these data, one can risk the statement that a contrast between high and low potential pulses (and thus ∆*P*) is less important than the steady current recovery. This statement can be also partially supported by the observation that the sample prepared under a larger U_H_-U_L_ contrast but lower anodizing temperature (the PAA—5 °C sample) is characterized by a comparable optical quality than the one fabricated under the lower U_H_-U_L_ contrast (the sample anodized under 40–20 V) but at higher anodizing temperature. In the latter case, however, most likely the higher temperature is the factor that improves structural (better regularity and circularity of pores) and thus optical properties PAA-based DBR.

## 4. Conclusions

In this work, the influence of anodization temperature from 5 °C to 30 °C range on the growth and photonic properties of PAA-based DBRs was studied. Transmission spectra were recorded to determine the position and a shape of PSBs. It was observed that above 10 °C and up to 25 °C the PSB properties strongly deteriorate as manifested in a progressive decrease, widening, and splitting of transmission peaks. However, at 30 °C the PSBs appeared again with several narrow, low intensity peaks in the 1000–2500 nm range and a strong, symmetric resonance peak in the MIR region. The photonic properties of the PAA—30 °C DBR were further improved by a small modification of the pulse sequence which sharpened the interface between d_H_ and d_L_ segments. Moreover, it was shown that larger U_H_-U_L_ contrast helps to increase the intensity of respective PSBs owing to the larger porosity contrast (∆*P*) between consecutive low and high RI layers. However, the larger U_H_-U_L_ contrast slowed down the current recovery (*i_a_^max^*) after application of subsequent U_H_ pulses. Generally, U_H_-U_L_ contrast seems to be less important than a steady *i_a_^max^* during application of consecutive pulses. Structural and optical properties of DBR anodized under 40–20 V at 10 °C were much better than the properties of the DBR synthesized under 50–30 V, despite the same U_H_-U_L_ contrast (20 V). On the other hand, the properties were comparable with that of the DBR synthesized under 50–20 V (U_H_-U_L_ contrast = 30 V), but at lower temperature of 5 °C. This means also that the temperature is an important factor in tailoring good quality PCs. Furthermore, the analysis performed in this work revealed that anodization at high temperature provides new conditions for designing and tailoring the PAA-based photonic structures with good photonic properties in MIR region. In fact, this was a first time the PAA-based DBR structure with a good quality PSB (relatively narrow and symmetric peak) in the MIR spectral range was fabricated. The new conditions provided by the high-temperature-pulse-anodization needs further optimization and mastering the electrochemical process (e.g., process conducted at T = 26–29 °C, various duration of U_H_ and U_L_ pulses, various U_H_-U_L_ contrast) in order to produce PAA-based photonic structure with excellent photonic properties (strong and narrow PSB resonances) in the MIR spectral range. This work is in progress.
